# Unsuspected Poisoning by Sterilized Meat and Milk of Tuberculous Animals*Paper presented at the Fourth Annual Meeting of the New York State Veterinary Medical Society.

**Published:** 1894-02

**Authors:** James Law

**Affiliations:** Cornell University, Ithaca, N. Y.


### UNSUSPECTED POISONING BY STERILIZED MEAT AND MILK OF TUBERCULOUS ANIMALS.*

BY JAMES LAW, F.R.C.V.S., Cornell University, Ithaca, N. Y.

With an unaccountable shortness of vision, medical and veterinary sanitarians alike have never, up to the present hour, looked beyond infection by the tubercle bacillus in estimating the dangers to man of tuberculosis in our flocks and herds. We find, accordingly, that the question kept continually before the public is that of the presence or absence of the tubercle bacillus in any food product—meat, milk, butter or cheese—furnished by the diseased or suspected animal. The question of the presence or absence of ptomaines or other toxic elements which are calculated to prove hurtful, or even fatal, to certain members of the human race is not for a moment considered.

Hence we are met by the most elaborate arguments that tubercle is rare in the muscular system of cattle, and that muscle juice is inimical to the bacillus, and that therefore the muscular tissue, which forms the great mass of the dressed carcass, may, as a rule, be safely eaten, even though the internal organs have been affected by tubercle.

In Germany and other European countries the flesh of animals in which the tubercles are found in only one organ, or two related ones, is passed as wholesome. It is only when the tubercles are found in the bones, or muscles, or in the lymphatic glands among these, or, finally, when the tubercles are so generally distributed in different parts of the body that it is evident that the bacilli must have been carried by the blood, that the meat is rejected as unfit for human food.

*Paper presented at the Fourth Annual Meeting of the New York State Veterinary Medical Society.

So with milk and other dairy products. Many claim with Nocard that the milk is quite harmless so long as the udder is quite free from tubercle, and that it is only when tubercle is unmistakably present in that gland that this secretion is to be dreaded.

Even apart from these discussions as to the wholesomeness of flesh and milk, it is safe to say that up to the present every writer on the subject holds that even tuberculous meat and milk is rendered absolutely harmless by cooking. The consensus of professional opinion on this subject is tersely given by Salmon and Smith in their article on "Tuberculosis," in the work on "Diseases of Cattle," published by the Bureau of Animal Industry—"Fortunately, tubercle bacilli are readily destroyed by the temperature of boiling water, and hence both meat and milk are made entirely safe, the former by the various processes of cooking, and the latter by boiling for a few minutes."

But this is altogether too narrow a view to take of the subject, and it is liable to lead to most serious and fatal results if put into every-day practice. The professional mind in concentrating its attention on *tubercular infection*, has practically entirely overlooked the no less real, and in many cases no less dangerous, fact of *tuberculous poisoning*.

To elucidate this matter, let us consider that much of the toxic matter produced by the growth of the tubercle bacillus is retained in Koch's "tuberculin," which has been absolutely sterilized. What then is the action of "tuberculin" on the animal system? A constitutional disorder with elevation of the body temperature commonly known as fever, and impairment of most of the bodily functions, notably those of assimilation and secretion. This is abundantly manifested in the wasting and fever of the victim of acute tuberculosis, in whom these poisonous principles are being constantly produced in large quantities. As the dose is reduced, a point is finally reached at which no fever nor appreciable systemic disturbance is produced, and thus in many slight and indolent cases of tuberculosis the animal appears well, and thus also the usual test dose of "tuberculin" has no recognizable disturbing effect on the healthy animal system. With a dose less than this it may even be questioned whether it may not be actually beneficial in conferring upon the healthy system a small measure of tolerance and power of resistance to the bacillus and its poisons. This, however, is of little account, seeing that no real immunity from tuberculosis is ever acquired. In many systems, both human and brute, the disease continues its slow progress for years, and the slight tolerance that results, though it may suppress the disease so that it assumes and indolent and chronic form, does not fully arrest it.

Very different is the effect of even a minimum dose of "tuberculin" on a subject which is already attacked with tuberculosis. In such a case the products of the existing tubercle are often so small in amount, and the system has acquired such a tolerance of them, that there is no manifest disturbance of health, and the animal may even be in excellent condition. But add to this minimum amount of poison, already in the system, a small quantity of "tuberculin," and in ten or fifteen hours the temperature of the patient's body will rise two or more degrees above the normal, and the destructive process going on in the seats of the tubercles will be accelerated. In cattle this is now used as a most valuable test of the presence or absence of occult tubercle. In horses and other animals, the subjects of tuberculosis, "tuberculin" causes the same rise of temperature, and it may be accepted as a rule applicable to all classes of animals.

In the tuberculous man this action of "tuberculin" is a well established fact and was made the basis of Koch's employment of this material as a curative agent. The daily use of "tuberculin" in cases of lupus and other superficial forms of tuberculosis led to a more active congestion and a molecular death of the tissues of the local tubercle, until these were separated from the living, healthy parts, and the progress of tuberculosis in that focus was arrested. If there were no deeper, unseen tubercles left in the system a real cure might be effected in this way. But the cure in such a case was only secured by a temporary aggravation of the disease in its primary focus. If other tubercles existed in internal organs, they too had the morbid process aggravated and extended, and the death of tissue increased, by the introduction of "tuberculin" from without. In such a case the increased mass of tubercle, dead and living, remained confined in the midst of the surrounding tissues, and as the infecting materials could not be separated and cast off from the body, they continued their ravages with an increasing force proportionate to their recent artificial extension.

It is this extension of the tuberculosis under the influence of the toxic products of the bacillus which raises the most important question in connection with the consumption by man of the flesh and dairy products of tuberculous animals, and yet this question has been overlooked by sanitarians in the most unaccountable way. It has seemed enough for them that the living tubercle bacillus did not exist in the juices of the muscles nor in the milk. It seems never to have occurred to them that all the soluble toxic products of this bacillus were constantly circulating in the blood passing through the muscles, and that they equally traversed the blood-vessels of the mammary glands and escaped into the milk. No pathologist can for a moment doubt this general diffusion of these toxic products.

Accepting this as undeniable, the presence of these toxins in the blood, flesh and milk, it follows that those who eat this meat and milk are taking in continually small doses of "tuberculin," and in case they are already the subjects of tuberculosis, in however slight a degree or however indolent a form, this continuous accession of the poison will rouse this morbid process into a greater activity and a more dangerous extension.

If now we consider the prevalence of tuberculosis in the human population, and realize that every eighth death is that of a tuberculous person, we see what a fearful risk is being run by the utilization of the meat and milk of tuberculous animals, even if it could be shown that such meat and milk are themselves free from the living bacillus. Such reckless consumption of the products of tuberculous animals can only be looked on as a direct means of sealing the fate of that large proportion of the community which is already slightly attacked with tuberculosis.

The claim that the canning of tuberculous carcasses and the boiling or Pasteurizing of milk does away with every element of danger, can no longer be entertained. Sterilization is not a restoration to a non-toxic condition; it does away with the possibility of infection, it is true, but it does not render the product innocuous.

As a matter of fact "tuberculin" has been sterilized by heat, but such sterilization has not rendered it safe and harmless. On the contrary it invariably intensifies any existing tuberculous process, and develops fever and constitutional disturbance. When "tuberculin," therefore, is present in the meat and milk, it can only cause these to operate in the same way on subjects that have been already infected. In my experience with tuberculous cows, cases have come to my knowledge in which invalids, drinking the milk of such animals, have suffered very obviously and have improved after such milk was withheld. So, too, in the case of calves sucking phthisical cows; they have done badly and proved unthrifty, though they took the whole of the milk furnished by their respective nurses, and they throve better when weaned and put upon solid food alone. I have followed some such calves until they grew up and were slaughtered and have made post-mortem examinations and found them bearing old calcified tubercles, pointing back to the time when they sucked the infected and poisonous milk.

It is idle to say that such milk was merely lacking in nutritive principles; the calves in question had access to other food while following their nurses, and would not have been harmed by taking the same amount of pure water as they did of milk. Apart from the bacilli, which operated slowly and which allowed these animals to live for years and even to thrive after they had ceased taking the milk, there was unquestionably in the milk a definite poison which undermined the health and stimulated the progress of the tuberculous process.

It may be safely held, as proved by analogy, observation and experiment, that the soluble poisons of tuberculosis invariably operate by exaggerating any existing tuberculous process, and that blood and all animal fluids becoming charged with such poisons always tend to further endanger the health, or even the life, of any person who may consume them while suffering from tuberculosis.

We may freely allow that the transmission of the bacillus from man to man is far more common than from beast to man. But though the implanted seed may have been in many cases derived from a fellow-man, its subsequent destructive progress may be due far more to the constant accessions of the soluble toxic products conveyed in the meat and milk of tuberculous animals. Without these constant doses of the soluble poisons of tubercle the implanted germ would in many cases have proved comparatively harmless. Although it could be proved in regard to many cases that the cow had not contributed the seed of the disease, she is left little less responsible for the destructive progress and fatal result. The germ which might have remained comparatively dormant and harmless in the absence of the poisoned meat and milk, is by these stimulated to a more deadly energy.

This hitherto unchallenged factor in the progress of tuberculosis opens up new and uncultivated fields for sanitary work. The great evil ventilated in this paper cannot be effectually met without the eradication of tuberculosis from every herd kept for supply of meat products for the public. Nothing short of this can be trusted to operate satisfactorily in putting a check upon the present fearful mortality from this disease. No inspection of dressed carcasses, nor of milk, butter and cheese will furnish a guarantee. We must go to the herds and subject them, animal by animal, to a critical test and only accept the products as safe when there is no longer a shadow of suspicion remaining.

A professional examination of the most searching kind must be supplemented by the tuberculin test before a clean bill of health can be furnished. In my own experience on cattle two-thirds of the cases of tuberculosis sometimes escaped under the most critical professional examination, and were detected later by the tuberculin test. Often when cattle were condemned by the "tuberculin" test, have the owners pronounced them the most thrifty and the least suspected in the herd, and it was only after death, when the animals were opened and the caseated tubercle exposed, that they were satisfied that no mistake had been made. Recently in a herd kept for the supply of high-priced milk of guaranteed soundness, the stock having been subjected to weekly examinations by a veterinarian, the "tuberculin" test was applied and 50 per cent. of the herd demonstrated to be tuberculous. Without the "tuberculin" test there is no guarantee possible for the products of the dairy, and the sanitary officers who will affect to deal with this disease in herds without the aid of "tuberculin" are at best but pruning the tips of the branches of the evil tree. Public money ought not to be thrown away on such fruitless and ineffective work. The purification of a herd must be followed in every case by a thorough disinfection of contaminated buildings and places, and by a careful seclusion from new sources of infection. It is evident, therefore, that the non-tuberculous herd must be secured against the addition of fresh animals from any herd that has not been similarly attested sound, and that any necessary addition from another source must be tested by "tuberculin" before it is added to the herd. Equally important is it to test all farm animals of whatever species that live on the place and cohabit with the herd, and to see to it that no human being suffering from tuberculosis is allowed to attend to the animals or to prepare their food. It is difficult to see how anything short of a system can afford any guarantee of the absence of the soluble tubercle poisons from our milk, butter and cheese.

In the case of butcher meats a professional examination when slaughtered, covering all of the viscera as well as the carcass, will be essential, and the current doctrine of sound meat with localized tuberculosis must be abandoned. Every municipality must have its own public abattoir, in which alone its meat supplies should be butchered and where every carcass should be systematically examined as it is opened. Private slaughter houses controlled by individual owners afford endless opportunities for the evasion of sanitary statutes and ought to be abandoned as relics of an age when modern sanitary science was unknown.

The question of dressed, canned and salted meats is one that must be carefully considered. It is quite evident that such products must come to us with a sufficient guarantee if allowed to compete with our home meats that have passed the municipal inspection. It is equally evident that no inspector, paid by the packer or canner, can furnish a certificate which will command public confidence. The inspector must be a government official who is entirely independent of the packers and whose position is in no way dependent on their good will. Then again the existing method of furnishing Government inspectors at our great packing centres only and thus giving a monopoly to the larger operators, cannot be long maintained in a country of equal rights and privileges. The most obvious cure for this evil is to make all packing establishments Government institutions, where the small packer shall have equal privileges with the large, and where all carcasses shall be subjected to the same scrutiny and shall go out with the same guarantee.

Such a proposition as the above will doubtless be severely criticised, both from the medical and economic standpoint.

On the medical side it will be argued that if the toxins in the meat and milk were as injurious as represented, we would see the evil results on every side and that medical men would be universally cognizant of them. And yet do we not see clearly to day much that was never suspected twenty, thirty, or fifty years ago? How recent is the acceptance by the profession of the doctrine of contagion in tuberculosis, in tetanus, in pneumonia, in influenza, in glanders, etc. Are we to suppose that our forefathers were surrounded with fewer evidences of contagion, at a time when no precautions were taken to prevent it, than we are with all the antiseptic and antizymodic provisions of the present day? The facts of contagion were doubtless more abundant in their than in these days, but their attention had never been drawn to them. So now let the attention of physicians and sanitarians be given to the morbid action of the soluble poisons of tubercle, and evidence of its evil will accumulate on all sides. It is the scrutiny and not the facts that are wanting.

The economist will object to drastic measures for the suppression of tuberculosis on the ground of expense. Who is to pay for the municipal abattoirs, the inspectorships, the disinfections and the indemnities for slaughtered animals and condemned carcasses? In return let us ask who now pays for the constant losses of live stock which the proposed system would put a stop to; for the frequent infection of sound herds by unfortunate purchases of animals that prove to be tuberculous; for the losses to the nation, the community and the family of the tuberculous one-eighth of all deaths; for the loss of work, literary, scientific, manufacturing, commercial, domestic and manual, of the great host of consumptives waiting all over the land to fill the places of this fatal eighth in coming mortality statistics; for the losses represented by the bills of the physician, nurse and druggist for these invalids; and for the losses represented by the many migrations and exiles in search of health, and of the costly consumption hospitals and sanitaria? And who is to pay in the future for the endless harvest of similar fruits which the seeds, now sown through our supineness, must inevitably produce in the coming generations?

Is it not a truer economy to destroy this seed before it has germinated, or even before it has been sown, than to wait for the multitudinous evils that must attend on its growth and fructification?

Our State Board of Health which has charge of tuberculosis, not in man only, but in cattle and other animals as well, should take this matter up earnestly. A year ago this Board was very apprehensive of evil results from the use of "tuberculin" as a means of diagnosis and only reluctantly allowed me to use the agent in examinations I made for it on condition I would personally bear the full responsibility for all untoward results. This I could afford to do, being well assured from past experience how trustworthy this agent is and how impossible it is to speedily eradicate tuberculosis from a herd without its aid.

Now that our excellent Board has reached a similar confidence, let me urge it to go a step farther, to accept the logical deduction of their present position, the presence, namely, of the toxic products in both meat and milk of the tuberculous animal and the pathogenic action of these on the tuberculous human consumer. Let our Health Board urge on the Legislature the necessity of providing means for the purification of our dairy herds and municipal abattoirs where all meats and animals for slaughter will be scrutinized by professional Government inspectors.

